# The role of temperamental self-regulation in predicting relapse in Alcohol Use Disorder

**DOI:** 10.1192/j.eurpsy.2023.719

**Published:** 2023-07-19

**Authors:** E. Santens, G. Dom, E. Dierckx, L. Claes

**Affiliations:** ^1^Addiction Psychiatry, Alexianen Zorggroep Tienen, Tienen; ^2^Psychiatry, University of Antwerp, Antwerp; ^3^Psychology, University of Brussels, Brussels; ^4^Psychology, KU Leuven, Leuven, Belgium

## Abstract

**Introduction:**

Alcohol Use Disorders (AUDs) are highly prevalent psychiatric disorders with often a poor treatment outcome in terms of high dropout rates and relapses. A vulnerability to disinhibition or a lack of self-regulation/low Effortful Control (EC) seems to be a core risk factor associated with both the initiation and continuation of AUDs. EC is the regulative dimension of temperament that involves attentional control, inhibitory control and activation control, and reflects self-regulation abilities that develop later in life parallel with the maturation of the prefrontal cortex.

**Objectives:**

In this study we want to investigate whether EC, operationalised in terms of self-report and in terms of behavioral measures, can predict relapse. When a low EC indeed turns out to be a significant contributor to relapse, treatment interventions aiming at strengthening EC could result in better treatment outcomes and less relapse in AUD patients.

**Methods:**

The sample consisted of 75 adult patients with AUD (68.9% males, mean age 47,4y) admitted at a specialized, inpatient treatment unit for addiction. To assess the regulative temperament dimension, we used the Effortful Control Scale (ECS) from the Adult Temperament Questionnaire Short-Form), a self-report questionnaire as well as five behavioral/neuropsychological tasks using the Cambridge Neuropsychological Test Automated Battery: Cambridge Gambling Task (CGT), Stop-Signal Task (SST), Intra-Extradimensional Set Shift (IED), Spatial Working Memory (SWM) and Spatial Span (SSP).

**Results:**

We performed binary logistic regression analyses with EC/CANTAB measures as predictors and relapse/no relapse (during treatment and after 3 months follow up) as dependent variable. According to these analyses, the self-report measure of EC nor the behavioral tasks CGT, SST, SWM and SSP (CANTAB) were able to significantly predict relapse neither during treatment nor after 3 months follow-up. Only the IED (outcome measure stages completed) was significant in predicting relapse (p<0.05) during follow-up.

**Conclusions:**

In our study we investigated whether self-regulation as measured by self-report questionnaires and behavioral tasks could predict relapse during treatment and after 3 months follow-up in a sample of inpatients with AUD. In contrast to some findings in literature, in our sample most of the used measures were not able to predict relapse. One hypothesis for these findings is that our sample of inpatients at a specialized addiction treatment unit is too homogeneous, all presenting lower levels of self-regulation. Future research should thus focus on larger samples and less homogeneous population. Only the IEC (outcome measure stages completed) was able to predict relapse during follow-up.

**Disclosure of Interest:**

None Declared

